# Aging During the Pandemic: Untangling the Complexities of COVID-19 and Geriatric Care

**DOI:** 10.14336/AD.2023.0405

**Published:** 2023-06-01

**Authors:** Kaimeng Su, Kunlin Jin

**Affiliations:** ^1^Grade 2021, clinical medicine 8-year program, Fudan University Shanghai Medical College, Shanghai, China; ^2^Department of Pharmacology and Neuroscience, University of North Texas Health Science Center, Fort Worth, TX 76107, USA

**Keywords:** COVID-19, pandemic, aging, health care, riskes, elderly

## Abstract

The COVID-19 pandemic has posed unprecedented challenges to the global healthcare system, with the elderly population being particularly vulnerable. This comprehensive review synthesizes the findings from publications in “*Aging and Disease*”, highlighting the unique challenges older adults encountered during the pandemic and providing solutions thereof. These studies provide invaluable insights into the elderly population’s vulnerabilities and needs during the COVID-19 pandemic. The susceptibility to the virus in older individuals remains debatable, and research on the clinical picture of COVID-19 in older populations has yielded insights into clinical features, molecular mechanisms, and potential therapeutic strategies. This review intends to shed light on the need of sustaining physical and mental well-being among older adults during the periods of lockdown by extensively exploring these concerns and emphasizing the need for targeted interventions and support systems for this population. Ultimately, the findings of these studies contribute to developing more effective and comprehensive approaches to managing and mitigating the risks posed by the pandemic to the elderly.

## Introduction

The COVID-19 pandemic has profoundly impacted global health, with older adults being one of the most vulnerable populations owing to age-related declines in immune function and increased prevalence of comorbidities. Immunosenescence or age-associated immune dysfunction contributes to their susceptibility to catastrophic consequences, such as greater morbidity and mortality rates, which makes it essential to identify specific preventative interventions for this demographic. Furthermore, in addition to biological factors, the psychosocial implications of the pandemic have significantly affected the well-being of older adults as they grapple with social isolation, disrupted healthcare services, and changes in lifestyle habits, which together poses additional health challenges. It is, therefore, imperative to explore the distinctive clinical manifestations, molecular underpinnings, and potential therapeutic strategies associated with COVID-19 in older adults to obtain data on evidence-based public health strategies, optimize resource distribution, and facilitate the development of targeted approaches for this at-risk group. The following studies published in “Aging and Disease” delve into various aspects of COVID-19 in older adults, shedding light on the complex interplay among age, immune function, and the disease course as well as exploring potential avenues for prevention, management, and treatment tailored to the needs of the elderly.

## Age-related factors, inflammation, immune response, and cognitive function

As the global population ages, understanding the complex interplay between aging, inflammation, and immune response in the context of COVID-19 infection becomes increasingly critical. It is critical to undertake research on the age-related factors that contribute to heightened vulnerability among elderly individuals, focusing on immune senescence, inflammation, and potential therapeutic targets. Thus, delving into the molecular mechanisms and clinical features of COVID-19 in older adults might offer insight into the need of establishing targeted interventions and strategies so as to improve the clinical management of this vulnerable population during the ongoing pandemic. The research findings demonstrate that older adults are more vulnerable to the virus due to age-induced alterations in immune functions, resulting in exacerbated inflammatory responses, increased disease severity, and psychological factors [1-6].

A comprehensive review of the clinical features of COVID-19 in elderly adults highlights that immunological senescence, defined as a loss in immune function with age, is a key factor leading to older adults’ increased susceptibility to the virus [3]. The authors discuss the molecular mechanisms of COVID-19 in elderly adults, including the role of angiotensin-converting enzyme 2 (ACE2) receptor expression that facilitates SARS-CoV-2 entry into host cells and cytokine storms, thereby contributing to the vulnerability of elderly individuals to the virus [3]. Similarly, Lara et al. hypothesize that NLRP3 inflammasome over-activation contributes to increased mortality rates in elderly patients with SARS-CoV-2 pneumonia [1]. This study highlights the importance of understanding the role of the NLRP3 inflammasome in COVID-19 and developing targeted therapies to regulate its activity in elderly patients [1].

Further, Zhang et al. explore the susceptibility of older adults to SARS-CoV-2 by evaluating the age-related differences in immune responses and clinical outcomes. This study reveals that older individuals exhibit a higher risk of severe COVID-19 infection and mortality owing to age-related declines in immune function [2]. These declines manifest as impaired antiviral defenses, reduced T-cell activity, and a heightened pro-inflammatory state, together contributing to increased disease severity in the elderly [2]. Similarly, Jain et al. delve into the contribution of immune senescence in the clinical pathophysiology of COVID-19 infection in the elderly [4]. The authors emphasize various mechanisms that contribute to immune senescence, including the decline of T-cell function and increased pro-inflammatory cytokines [4]. Pre-existing comorbidities such as diabetes and cardiovascular disease are also mentioned as potentially exacerbating immune senescence and worsening COVID-19 outcomes [4]. To summarize, Moraes et al. employ a transcriptomic approach to predict SARS-CoV-2 interaction with host proteins during lung aging, revealing a potential role for the protein tribbles pseudokinase 3 (TRIB3) in COVID-19 [5]. According to these findings, TRIB3, a protein regulating cellular metabolism and inflammation, is a potential mediator of viral entry and replication; thus, targeting TRIB3 might be a potential therapeutic strategy for COVID-19, especially in older individuals or those with comorbidities [5].

Notably, the COVID-19 pandemic has not only affected the lives of older adults, especially those suffering from cognitive impairments and dementia physically but also has had a profound and significant impact on the psychological factors. Understanding the impact of the pandemic risk on brain health and cognitive function is critical as the threat spreads. Consequently, it is critical to focus on understanding the intricacies of how COVID-19 affects cognitive function and dementia in older individuals, shedding light on the potential risk factors, exacerbation of symptoms, and the need for tailored care approaches. COVID-19 may induce cognitive impairment, such as inflammation, hypoxia, and endothelial dysfunction. Rizzo and Paolisso suggest that the virus may exacerbate or accelerate cognitive decline, especially in those with pre-existing neurodegenerative disorders, while stressing the need for targeted interventions to mitigate these effects [6]. These findings underscore the importance of understanding the unique challenges faced by this vulnerable population and developing targeted preventative and management strategies to mitigate the virus’s long-term consequences on cognitive functioning and the overall quality of life.

## COVID-19 and its impact on specific geriatric conditions and lifestyle factors

Due to age-related physiological changes, comorbidities, and impaired immune function, the COVID-19 pandemic has disproportionately affected older adults, who are at a higher risk of catastrophic consequences. While the significant emphasis has been made on the general consequences of COVID-19 on the elderly, it is equally important to explore the virus's impact on specific geriatric conditions during lockdowns. Such investigations would provide valuable insights to address the unique needs of the elderly population during the pandemic.

SARS-CoV-2 infection increases the risk of bone loss and resorption in the elderly. Tang opines that the pandemic's influence on lifestyle, healthcare, and treatment accessibility has raised the incidence of osteoporosis in older adults [7]. According to the author, lockdown measures, social isolation, and decreased physical activity have all contributed to the deterioration of bone health in the elderly [7]. In addition, the pandemic has limited the access to healthcare services, such as routine check-ups and diagnostic tests, thereby further exacerbating the issue. The author also emphasizes the connections among COVID-19, glucocorticoids, and osteoporosis in elderly patients, including stress, altered sleeping patterns, and poor nutrition [7]. Furthermore, a systematic review of the effect of COVID-19-related lockdowns on diet and physical activity in older adults reveals that lockdowns causes a decline in physical activity and deterioration of dietary habits in this population [8]. As both exercise and nutrition are essential for maintaining overall health, especially for the elderly, the study emphasizes the need for effective strategies to counteract these negative consequences of lockdowns [8]. Thus, regular physical activity to maintain a healthy immune system is frequently advised, notably to the elderly during the COVID-19 pandemic. Regular physical activity boosts immunity, reduces inflammation, and improves vaccination efficacy. It is also recommended that older adults engage in moderate-intensity exercise tailored to individual needs and the abilities to enhance their immune response and potentially mitigate the adverse effects of COVID-19 [9].

Men are at a higher risk of severe illness and mortality, whereas women are more likely to experience mild symptoms, suggesting that sex hormones may play a role in this disparity. Okpechi et al. describe how sex hormones affect the immune system and how this may contribute to the observed sex differences from COVID-19. Moreover, the authors suggest that hormone replacement therapy (HRT) might be a viable therapeutic option for older adults, particularly women, who are at higher risk for severe COVID-19 outcomes [10]. They suggest that HRT may help modulate the immune response to COVID-19 owing to its anti-inflammatory characteristics. However, further research is warranted to determine the efficacy and safety of HRT in the context of COVID-19 [10]. These studies highlight the myriad ways in which COVID-19 affects specific geriatric conditions, underlining the importance of a comprehensive understanding of the virus's impact on older adults.

## COVID-19 clinical features and management in elderly populations

As the world grapples with the ongoing COVID-19 pandemic crisis, it is crucial to understand the unique challenges that older adults face and, accordingly, develop targeted strategies to improve their health outcomes. A review of research on the clinical features, susceptibility, and management of COVID-19 in elderly populations has underscored the importance of considering age-related factors, such as frailty and immune dysfunction, in guiding clinical care and resource allocation. They also highlight the need for targeted prevention and management strategies, such as prioritizing vaccinations and integrating telemedicine, so as to ensure the well-being of older adults during these unprecedented times [11-15].

A qualitative systematic review of the literature published up to June 2020, focusing on studies that reported on clinical features, risk factors, and outcomes of COVID-19 in patients aged 60 years and above, highlights that elderly patients with COVID-19 are more likely than younger patients to present with atypical symptoms, such as delirium, fatigue, and anorexia [14]. Given their heightened sensitivity and the risk of poor outcomes, the authors emphasize the need for timely and appropriate management in elderly patients with COVID-19 [14]. The review suggests that early identification, monitoring, and treatment of comorbidities may improve the clinical outcomes in this population [14]. Overall, the review emphasizes the importance of recognizing the unique clinical characteristics and risk factors associated with COVID-19 in the elderly population, as well as the necessity for tailored approaches to manage and treat COVID-19 in this vulnerable group [14]. Jankowska-Polańska et al. have conducted additional research into the unique challenges faced by elderly patients during the pandemic, including those with dementia, and proposed that telemedicine, remote patient monitoring, and digital health solutions can help maintain continuity of care for these individuals [11]. The authors highlight the need for healthcare personnel to adapt to the new realities of caring for elderly patients during the pandemic and further suggest that, by implementing appropriate measures, healthcare personnel can ensure that elderly patients receive the necessary care while minimizing the risk of transmission [11].

An interesting report on the impact of the COVID-19 pandemic on the medical services for elderly patients in China highlights disruptions in healthcare delivery, such as fewer outpatient visits and delayed treatments, which disproportionately affect older adults with chronic conditions [13]. The authors urge that during the pandemic, policymakers and healthcare providers prioritize the needs of the elderly population, such as through implementing telemedicine services and ensuring the availability of necessary medical supplies [13]. Similarly, Lv and Yin have reviewed the experiences of patients with dementia living at home in China during the COVID-19 pandemic and provided recommendations for preventing and managing COVID-19 [12]. They highlight the need for tailored prevention and management strategies, including providing personal protective equipment, telemedicine, and home-based care services, so as to ensure the safety and well-being of dementia patients and caregivers [12]. A multicentre study conducted during the first two pandemic waves in Italy to explore the role of age and frailty in the outcomes of mechanically ventilated COVID-19 patients reveals that frailty, rather than chronological age, is a significant predictor of poor outcomes in mechanically ventilated patients [15]. This study highlights the importance of considering frailty status in clinical decision-making and resource allocation during a pandemic.

Above all, it is critical to comprehend the potential end of the COVID-19 pandemic. Previous pandemics have concluded that pandemics typically terminate when the majority of the population achieves herd immunity through natural infections or vaccinations. The most effective strategy to achieve herd immunity against COVID-19 would be a worldwide immunization program. To improve immune functions in older people, targeted interventions, such as vaccination strategies and treatments that can enhance immunological responses are required [4]. Continued vigilance and the necessity for governments and individuals to maintain preventative measures, including wearing masks, social distancing, and proper hygiene are critical until herd immunity is achieved. Older individuals, especially those with comorbidities, are at a higher risk of severe outcomes and should be prioritized for vaccination to reduce morbidity and mortality. Furthermore, there is a need for equitable vaccine distribution and global cooperation to safeguard vulnerable populations [16]. In fact, monitoring the disease's progression in different age groups, particularly among the elderly, should be better comprehended to manage the pandemic [17]. These studies highlight the importance of timely vaccination, improved geriatric medical services, and the adoption of innovative care strategies. Healthcare professionals and policymakers can ensure that the well-being of elderly populations is prioritized during these unprecedented times by recognizing and addressing these issues.

## Conclusion

The elderly are disproportionately impacted by COVID-19, with higher risks of related severe illness, hospitalization, and mortality. A comprehensive understanding of the factors contributing to this increased vulnerability and the impact of the pandemic on various aspects of geriatric health is therefore essential for designing effective prevention and management strategies. The studies compiled in “*Aging and Disease*” provide a holistic overview of the challenges faced by older adults during the pandemic and the potential interventions to alleviate these risks. The knowledge gained thereby can raise awareness and provide information to healthcare professionals, policymakers, and caregivers on evidence-based approaches to support the well-being of the elderly population in the context of the COVID-19 pandemic and future public health emergencies.
